# Massive Retroperitoneal Hematoma Following Vaginal Delivery: A Case Report and Review of Literature

**DOI:** 10.7759/cureus.102505

**Published:** 2026-01-28

**Authors:** Ali El Safadi, Marwa Obeid, Nathalie Chamseddine, Ihab Usta

**Affiliations:** 1 Obstetrics and Gynecology, American University of Beirut Medical Center, Beirut, LBN

**Keywords:** episiotomy, major trauma, postpartum hemorrhage, retroperitoneal hematoma, vaginal delivery

## Abstract

Retroperitoneal hematoma is a rare but potentially life-threatening cause of concealed postpartum hemorrhage. Because bleeding is contained within the retroperitoneum, external blood loss may be absent, and symptoms may be nonspecific, resulting in delayed diagnosis. Rapid recognition and intervention are essential to reduce morbidity and mortality. We report a 21-year-old primigravida who developed severe hypotension and pelvic pain a few hours after an uncomplicated vaginal delivery with a small median episiotomy. Ultrasound revealed a 9 × 9 cm pelvic hematoma. Despite aggressive resuscitation, she required laparotomy, which demonstrated diffuse venous oozing from the retro pubic region without an arterial source. Hemostasis was achieved with combined vaginal and retroperitoneal packing. She required a total of 23 units of packed red blood cells, 18 units of fresh frozen plasma, five units of platelets, and two units of cryoprecipitate. She fully recovered following staged re-exploration and definitive closure. Retroperitoneal hematoma should be suspected in postpartum patients with hemodynamic instability and no external bleeding. A multidisciplinary approach and individualized management, including surgical packing, are critical for successful outcomes.

## Introduction

Retroperitoneal hematoma in the postpartum period is a rare but serious complication of vaginal delivery. The estimated incidence ranges from 1 in 300 to 1 in 1500 births, although the true incidence is likely underestimated due to under-reporting and variable severity [1]. The retroperitoneal space can accommodate large volumes of blood before clinical signs become apparent, making this condition a concealed and particularly dangerous form of postpartum hemorrhage.

These hematomas typically result from injury to branches of the internal iliac system, including the uterine, vaginal, pudendal, or obturator arteries, or from disruption of richly anastomotic venous plexuses, particularly the paravaginal venous network [2]. Venous bleeding, although slower than arterial bleeding, can be extensive and may occur without a clear focal source. Radial stretching of the birth canal during parturition can cause contusion or avulsion of the vascular supply without disrupting the overlying epithelium [3].

Reported risk factors include episiotomy, perineal lacerations, prolonged or precipitous labor, nulliparity or multiparity, macrosomia, multiple pregnancy, hypertensive disorders including preeclampsia and Hemolysis, Elevated Liver enzymes and Low Platelets (HELLP) syndrome, operative vaginal delivery, manual removal of the placenta, and rarely underlying connective tissue or coagulation disorders [4]. However, retroperitoneal hematomas have also been described after uncomplicated vaginal deliveries without identifiable trauma, underscoring the need for a high index of suspicion [5].

Clinical presentation is often subtle and evolves over time. Early symptoms such as pelvic or flank pain, urinary retention, or mild anemia may be misattributed to routine postpartum changes. Hemodynamic instability usually occurs only after substantial concealed blood loss. Because external bleeding is often absent, imaging plays a key role in diagnosis. While ultrasound may identify pelvic collections, it may miss retroperitoneal extension; computed tomography (CT) angiography remains the diagnostic modality of choice in stable patients [6].

Management depends on hemodynamic status and the bleeding source. Conservative management may be appropriate for stable patients with non-expanding hematomas. Interventional radiology can be effective when arterial extravasation is identified. In unstable patients, immediate surgical intervention, including hematoma evacuation, vessel ligation, retroperitoneal packing, or hysterectomy in extreme cases, may be required [7]. We aim to highlight the importance of early recognition and multidisciplinary management of retroperitoneal hematoma as a rare, life-threatening cause of concealed postpartum hemorrhage.

## Case presentation

A 21-year-old primigravida at 39 weeks and five days of gestation presented in active labor with regular uterine contractions. Labor progressed spontaneously; the duration of first stage was eight hours and 53 minutes, the second stage of labor was 57 minutes, and the third stage of labor was one minute. A living female infant, weighing 2910 grams, Apgar scores 8 and 9 at one and five minutes, was delivered vaginally and did not require operative intervention. A small median episiotomy was performed. Inspection revealed a second-degree perineal and vaginal laceration similar to a right mediolateral episiotomy. Bleeding was noted at the apex of the laceration which resolved with suturing beyond the apex and repairing in layers. The episiotomy was also repaired in layers, and a vaginal pack was placed for hemostasis because of friable vaginal mucosa, which was removed after two hours with no noticeable vaginal bleeding.

**Figure 1:**
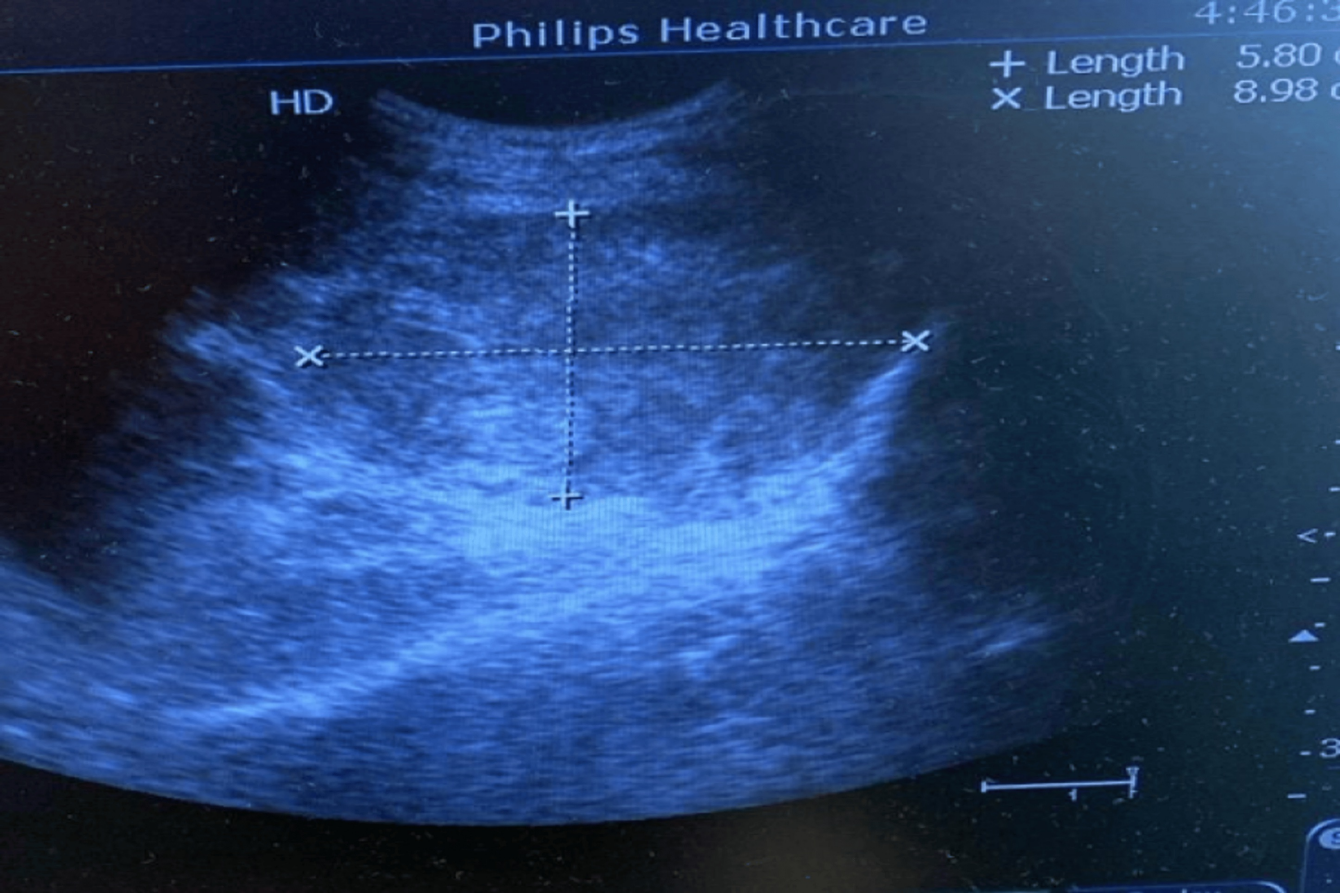
Bedside ultrasound image demonstrating a large pelvic hematoma measuring 9 × 5.8 cm (postpartum).

Approximately one hour later, the patient complained of severe suprapubic and pelvic pain, urinary retention (450 mL drained via catheterization), and dyspnea. A rapidly worsening pallor and hypotension (50-60/40 mmHg) were noted. She had no uterine atony or active vaginal bleeding, and perineal examination revealed no rectovaginal collection or perineal ecchymosis. Two large-bore IV lines were inserted, and the rapid response team was called. CBC was requested and bedside ultrasound revealed a 9 × 5.8 cm pelvic hematoma in the right prescaral area, shifting the uterus and bladder to the left as seen in Figure 1. Hemoglobin had dropped from 15 g/dL to 9.4 g/dL. Given her clinical deterioration, she was transferred emergently to the operating room. Intraoperative ultrasound demonstrated interval expansion of the hematoma to 10 × 6 cm.

In the operating room, the vaginal laceration was opened and large clots were removed; it was clear that the active bleeding was from beyond the apex of the laceration, inaccessible vaginally, and a tunnel extending into the paravaginal/retroperitoneal space was palpable. The laceration was re-sutured. At this time, friable tissues and diffuse oozing suggestive of disseminated intravascular coagulopathy (DIC) was noted and a vaginal pack was inserted.

Given hemodynamic instability and ongoing hemorrhage, a midline exploratory laparotomy was performed in collaboration with obstetrics, gynecologic oncology, general surgery, and vascular surgery teams. Upon entry, hemoperitoneum and retroperitoneal hematomas were evacuated. Large clots were infiltrating the anterior abdominal muscles. The retroperitoneal space was opened and no discrete arterial source was identified; instead, diffuse venous oozing was noted. Hemostasis was partially achieved with sutures and hemostatic agents (Gelfoam™ (Pfizer, New York, NY, USA), Surgicel™, and Fibrillar™ (both from Ethicon, Raritan, NJ, USA)). Because of marked tissue friability and coagulopathy, retroperitoneal and vaginal packing was required (five abdominal pads and one vaginal pad). The fascia was left open with temporary skin closure. Intraoperatively, the patient received 15 units of packed red blood cells (pRBCs), 15 units of fresh frozen plasma (FFP), three platelet units, one unit of cryoprecipitate, and two doses of tranexamic acid. Estimated blood loss was 8 liters. She was transferred intubated to the surgical intensive care unit.

In the surgical unit, she required an additional six units of pRBCs, seven units of FFP, and two platelet units, for a total of 23 pRBCs, 18 FFP, five platelets, and two cryoprecipitate. Hemoglobin stabilized at 8.5 g/dL and electrolyte imbalance was corrected. Urine output was good throughout. The DIC panel returned negative except for thrombocytopenia (58,000/µL), low fibrinogen, mild increase in international normalised ratio (INR) level, and high D-dimer level as seen in Table 1. Urine culture grew Streptococcus agalactiae, prompting escalation of antibiotics to piperacillin-tazobactam (4.5 g every six hours). Lower-extremity Doppler ultrasound excluded deep venous thrombosis (DVT). CT angiography showed no active bleeding.

**Table 1:** Serial Hematologic Parameters During the Clinical Course INR: international normalised ratio

Parameter	Reference Range	Pre-delivery	Postpartum	Post-operative / ICU admission	During ICU stay
Hemoglobin (g/dL)	12.0 – 16.0	15.0	9.4	8.5	9.0
Hematocrit (%)	36 – 46	43	27	24	25
Platelets (×10³/µL)	150 – 400	152	173	56	58
INR	0.9 – 1.1	—	1.2	1.2 – 1.4	1.0 – 1.1
Prothrombin Time (s)	10 – 13	—	13.2	14.9 – 15.1	10.8 – 11.8
Fibrinogen (g/L)	1.7 – 4.0	—	2.36	1.24	1.72 – 2.81
D-dimer (ng/mL)	< 243	—	2,759	254 – 292	—

On postoperative day (POD) three, she underwent planned re-exploration and fascial closure. No active bleeding was identified. All abdominal and vaginal packs were removed, the cavities irrigated with saline containing gentamicin and vancomycin, and bilateral Jackson-Pratt drains were placed. She tolerated the procedure well and was extubated thereafter.

Following the second-look laparotomy, the patient remained in the surgical intensive care unit for one day. A postoperative chest radiograph showed mild pulmonary congestion, which improved with a single dose of furosemide. She continued on total parenteral nutrition and gradually transitioned to oral intake. Cultures obtained from the abdominal pads and hemostatic agents later grew extended-spectrum beta-lactamase (ESBL)-producing Escherichia coli and Candida albicans, although blood, urine, and tracheal aspirate cultures initially showed no growth.

Once clinically stable, she was transferred to the regular floor. Her nasogastric tube, Foley catheter, surgical drains, and central venous line were removed sequentially. She ambulated, resumed breast pumping, and tolerated her diet. Her surgical wound remained clean with daily dressing care.

During her stay, she initially experienced intermittent low-grade fevers that were attributed to breast engorgement but later developed high-grade fevers and tachycardia. The Infectious Diseases team discontinued piperacillin-tazobactam and initiated meropenem, along with caspofungin in response to Candida albicans growth from abdominal cultures. Broad infectious workup including repeat blood and urine cultures, sputum culture, cytomegalovirus (CMV) PCR, triple respiratory PCR, and imaging revealed moderate Klebsiella pneumoniae in sputum and ESBL-producing E. coli and Candida albicans from abdominal materials, while all blood cultures remained negative. She also developed an erythematous papular rash over the shins and buttocks. Dermatology diagnosed cutaneous candidiasis on potassium hydroxide (KOH) smear. Topical antifungal therapy was added to the caspofungin.

Her fevers gradually resolved, and she remained afebrile for four days. With sustained clinical improvement, she was discharged home on a seven-day course of oral trimethoprim-sulfamethoxazole and a 14-day course of oral fluconazole, along with enoxaparin for thromboprophylaxis. An Edinburgh Postnatal Depression Scale score of 14 was documented; however, her affect was appropriate, and she was counseled on postpartum mental health and follow-up.

## Discussion

Retroperitoneal hematoma following vaginal delivery is an uncommon but potentially life-threatening cause of concealed postpartum hemorrhage. Its clinical significance lies in the fact that bleeding occurs in a non-visible compartment and is often recognized only after the patient becomes hemodynamically unstable. Hemorrhage may originate from arterial branches of the internal iliac system; however, more frequently it results from disruption of the paravaginal venous plexus, a highly distensible and richly anastomotic network that is particularly vulnerable to stretching and shearing forces during childbirth [2,3]. In the present case, surgical exploration revealed diffuse venous oozing without an identifiable arterial source, a pattern consistent with previously reported venous plexus injuries rather than focal vascular disruption.

This case underscores that even seemingly minor perineal trauma can permit extension of bleeding into the paravaginal and retroperitoneal spaces. Although classical risk factors include episiotomy, perineal lacerations, operative vaginal delivery, prolonged or precipitous labor, hypertensive disorders of pregnancy, and coagulation abnormalities, retroperitoneal hematomas have also been described following uncomplicated spontaneous vaginal deliveries with no instrumentation [5]. This highlights the importance of maintaining a high index of suspicion regardless of perceived obstetric risk.

The anatomic level of the hematoma plays a critical role in both clinical presentation and detection. Hematomas located below the levator ani muscle typically extend laterally toward the perineum and rectum and may present as a tense, fluctuant, and exquisitely painful vaginal or perineal mass. In contrast, hematomas located above the levator ani may extend toward the broad ligament or retroperitoneal space, often without obvious vaginal or perineal findings, as occurred in our patient [6]. In such cases, pain out of proportion to physical examination, particularly when associated with urinary retention, anemia, or hypotension, should prompt immediate investigation for concealed hemorrhage.

Diagnosis remains challenging because early clinical signs are nonspecific and routine pelvic examination may be unrevealing. Bedside ultrasound can identify pelvic collections but may underestimate the extent of retroperitoneal involvement. In hemodynamically stable patients with unclear findings, computed tomography angiography is the preferred imaging modality, as it allows accurate delineation of hematoma extent and identification of active bleeding [6]. Massive concealed blood loss may precipitate consumptive coagulopathy, as transiently observed in our patient, underscoring the need for early laboratory assessment and prompt initiation of massive transfusion protocols. Management strategies must be individualized based on hemodynamic status, hematoma progression, and bleeding source. Conservative management may be appropriate for stable patients with non-expanding hematomas, as demonstrated in several reported cases [4,5,8]. Interventional radiology with selective embolization is highly effective when arterial extravasation is identified and the patient is sufficiently stable [9]. However, in rapidly deteriorating patients or in cases of diffuse venous bleeding without a focal source, embolization may not be feasible. In such circumstances, surgical intervention with retroperitoneal and vaginal packing represents a lifesaving temporizing strategy [2,7].

As summarized in Table 2, previously reported cases demonstrate considerable heterogeneity in etiology, presentation, imaging findings, and management approaches. While most patients ultimately recover, severe cases often require aggressive surgical management and massive transfusion, as in the present case. Our experience supports existing evidence that combined vaginal and retroperitoneal packing is an effective strategy when conventional suturing and hemostatic measures fail.

**Table 2:** Reported cases of postpartum retroperitoneal hematoma following vaginal delivery (2005–2025) CT: computed tomography; NR: not required; US: ultrasound; PPH: postpartum hemorrhage; SVD: spontaneous vaginal delivery; RPH: retroperitoneal hematoma; PRBC: packed red blood cells; FFP: fresh frozen plasma

Author (Year)	Delivery	Trigger / Etiology	Presentation	Imaging	Management	Transfusion / Intervention	Outcome
Fieni et al. (2005) [10]	SVD	Retzius space hematoma	Pelvic pain, urinary retention	Ultrasound	Conservative	NR	Recovered
Rafi & Muppala (2008) [5]	SVD	Spontaneous psoas hematoma	Pain, anemia	NR	Conservative	NR	Recovered
Singh et al. (2008) [3]	SVD	Ischiorectal/retroperitoneal hematoma	Pain ± instability	NR	NR	NR	Recovered
Acreman & Sainani (2018) [7]	SVD	Retzius space hematoma	Concealed PPH	CT/US	Individualized	NR	Recovered
Maroyi et al. (2021) [2]	SVD	Large retroperitoneal hematoma	Delayed diagnosis	CT	Surgical/Conservative	NR	Recovered
Zon et al. (2022) [8]	SVD	Retzius space hematoma	Pelvic pain	CT/US	Conservative	NR	Recovered
Villatoro et al. (2022) [6]	Instrumental VD	Traumatic RPH	Pain, anemia, shock	CT	Mixed	NR	Recovered
Haddadi et al. (2024) [4]	SVD + episiotomy	Expanding pelvic RPH	Pelvic/abdominal pain	CT	Conservative	NR	Recovered
Rodrigo & Chuang (2025) [9]	SVD	Ovarian artery rupture	Hemorrhagic shock	CT angiography	Angioembolization	IR embolization	Recovered
Present case (2025)	SVD + median episiotomy	Diffuse venous retroperitoneal bleeding	Severe pelvic pain, urinary retention, shock	US, CT angiography	Exploratory laparotomy + retroperitoneal & vaginal packing	Massive transfusion (23 PRBC, 18 FFP)	Recovered

A notable aspect of this case was the development of postoperative infectious complications, including ESBL-producing Escherichia coli and Candida albicans cultured from packing materials. Prolonged packing, devitalized tissue, and broad-spectrum antibiotic exposure likely contributed to microbial proliferation. These complications emphasize the importance of planned re-exploration for pack removal once hemostasis is achieved and close collaboration with infectious disease specialists in the postoperative period.

Despite massive transfusion requirements and a prolonged intensive care and hospital course, the patient recovered fully without long-term sequelae. This case illustrates the importance of early recognition of concealed postpartum hemorrhage, rapid multidisciplinary coordination, and flexibility in selecting hemostatic strategies tailored to the bleeding pattern.

This case represents an unusual event having a massive concealed retroperitoneal hematoma following an apparently uncomplicated vaginal delivery, with no external bleeding or uterine atony. It highlights the diagnostic challenge posed by nonspecific early symptoms progressing rapidly to profound hemorrhagic shock. Intraoperatively, bleeding was diffuse and venous without an identifiable arterial source, requiring combined vaginal and retroperitoneal packing with staged laparotomy. The case underscores the value of early bedside ultrasound and multidisciplinary management in achieving survival despite extreme transfusion requirements.

## Conclusions

Retroperitoneal hematoma should be considered in postpartum women with unexplained pelvic pain, anemia, or hemodynamic instability in the absence of visible bleeding. Prompt imaging, aggressive resuscitation, and coordinated multidisciplinary management, including surgical exploration and packing when indicated, are essential for favorable maternal outcomes. Future research should focus on refining diagnostic pathways and management strategies, particularly the role of early bedside imaging, while multicenter studies are needed to better define risk factors, transfusion needs, and standardized treatment approaches for this rare but severe condition.

## References

[1] Nassar A, Kuntal S, Parveen S (2016). Spontaneous retroperitoneal haematoma post spontaneous vaginal delivery: a case report. Eur J Obstet Gynecol Reprod Biol.

[2] Maroyi R, Ngeleza N, Kalunga K, Buhendwa C, Shahid U, Boij R, Mukwege D (2021). Large retroperitoneal hematoma following vaginal delivery: a case report. J Med Case Rep.

[3] Singh J, Basu S, Aich A, Bhal PS (2008). Spontaneous ischiorectal and retro-peritoneal haematoma after normal vaginal delivery. J Obstet Gynaecol.

[4] Haddadi M, Hantoushzadeh S, Pesikhani MD, Asadi F, Amini S, Ghaemi M (2024). Conservative management of retroperitoneal hematoma expanded to prerenal space due to episiotomy in a woman with vaginal delivery: a case report. Int J Surg Case Rep.

[5] Rafi J, Muppala H (2008). Conservative management of massive puerperal spontaneous onset retroperitoneal psoas muscle haematoma following normal vaginal delivery. J Obstet Gynaecol.

[6] Redondo Villatoro A, Azcona Sutil L, Vargas Gálvez D, Carmona Domínguez E, Cabezas Palacios MN (2022). Diagnosis and management of postpartum retroperitoneal hematoma: a report of three cases. Am J Case Rep.

[7] Acreman ML, Sainani M (2018). Retzius space haematoma as a rare cause of concealed retroperitoneal postpartum haemorrhage following spontaneous vaginal delivery. BMJ Case Rep.

[8] Zon EM, Afendi NR, Ismail MP, Ibrahim A, Che Hashim NA (2022). Conservative management of Retzius space hematoma following spontaneous vaginal delivery in a woman with an unscarred uterus: a case report. Case Rep Womens Health.

[9] Rodrigo V, Chuang TY (2025). A rare presentation of retroperitoneal hemorrhage following a spontaneous vaginal delivery. Cureus.

[10] Fieni S, Berretta R, Merisio C, Melpignano M, Gramellini D (2005). Retzius' space haematoma after spontaneous delivery: a case report. Acta Biomed.

